# MMP-9 and CCR7 as Possible Predictors of Lymph Node Metastasis in Laryngeal Squamous Cell Carcinoma

**DOI:** 10.30699/ijp.2023.563014.2986

**Published:** 2023-06-20

**Authors:** Lisnawati Rachmadi, Ela Laelasari, Yayi Dwina Billianti Susanto, Kusmardi Kusmardi

**Affiliations:** *Department of Anatomical Pathology, Faculty of Medicine, Universitas Indonesia* */Cipto Mangunkusumo Hospital, Jakarta, * *Indonesia*

**Keywords:** Laryngeal Squamous Carcinoma, MMP-9, CCR7, Lymph Node Metastasis

## Abstract

**Background & Objective:**

The expression of matrix metalloproteinase-9 (MMP-9) and chemokine receptor 7 (CCR7) is significantly associated with tumor invasion and metastasis. Little is known regarding the potential of these markers in predicting cancer metastasis in Laryngeal Squamous Cell Carcinoma (LSCC). Therefore, this study aimed to dissect the potential of these markers in predicting the lymph node metastasis in LSCC patients.

**Methods:**

Sixty tissue samples were obtained from the patients diagnosed pathologically with LSCC who underwent partial or total laryngectomy. The expression of MMP-9 and CCR7 was measured using the immunohistochemistry staining in the tissue samples of LSCC patients. The ROC (receiver operating characteristic) curve was used to determine the most significant cut-off points of expression according to the highest sensitivity and specificity of both the markers to predict the lymph node metastasis in LSCC. Then, the relationship between the clinicopathology features and the expression of MMP-9 and CCR7 was evaluated.

**Results:**

The expression of both MMP-9 and CCR7 was significantly correlated with the lymph node metastasis in LSCC (*P*<0.001). Furthermore, CCR7 expression exhibited the highest prediction accuracy (AUC 95.7%) and sensitivity (100%) in predicting the lymph node metastasis in LSCC compared to that of MMP-9 (AUC 92.9%, sensitivity 90%). We also found that patients with larger tumor size (> 4 cm) had significantly higher expression of MMP-9 and CCR7 (*P*<0.002 and *P*<0.001, respectively). The Elevated expression level of CCR7 statistically correlated with higher MMP-9 expression (*P*<0.001).

**Conclusion:**

MMP-9 and CCR7 might be beneficial as predictors of lymph node metastasis in LSCC patients.

## Introduction

Laryngeal squamous cell carcinoma (LSCC) has become the second most prevalent malignant tumor of the respiratory tract and of head and neck (1). It is estimated that LSCC incidence was 2.76 cases/year per 100 000 inhabitants, and more than a large majority of malignancies in the larynx are detected as squamous cell carcinomas (2, 3).

The surgical intervention followed by radiotherapy and chemotherapy remains the standard approach for treating LSCC; these modalities, however, commonly result in a poor prognosis for a patient in the advanced stage of LSCC(4-7). Thus, dissecting the molecular mechanisms associated with the carcinogenesis of LSCC is essential for identifying markers that help establish an appropriate patient treatment(8).

Lymph node metastasis provides information from a serious prognostic sign associated with treating the LSCC patients(9). The cervical lymph nodes are the primary sites of metastasis in LSCC, followed by distant metastases(10). Cancer metastasis is an intricate process, namely the separation of unstable cells from other cells in the first step. Subsequently, the migration of tumor cells occurs based on the proteolytic enzyme that degrades the extracellular matrix and basement membrane so that the tumor cells are able to invade and metastasize into lymphovascular and other organs(11, 12). 

Matrix metalloproteinase-9 (MMP-9) has an important role in the degradation process of the extracellular matrix and basement membrane, as well as regulating the activity of several growth factors and cytokines (13). This action affects the immune response, angiogenesis, tumor cell proliferation, and metastasis (11). The spread of tumor through lymphatic pathways and metastasis to lymph nodes were associated with the increased expression of MMP-9 (14). A previous study revealed that MMP-9 expression was associated with poorly differentiated tumors along with lymph node metastasis. It could also help in recognizing the presence of occult metastasis, and serve as a prognostic marker in LSCC (10, 13). MMP-9 activation enhances the chemotaxis and migration of LSCC tumor cells to cervical lymph nodes which is mediated by chemokine receptor 7 (CCR7) (15). Its expression also played a role in tumor cell metastasis to neck lymph nodes and was mediated by dendritic cells (16). CCR7 was a chemokine receptor that played a role as a chemoattractant binding to its ligands and mediated the metastasis of cervical lymph nodes (17). Increased expression of CCR7 is directly related to the incidence of metastases to cervical lymph nodes and tumor progression (18). This retrospective study was designed to underpin the importance of MMP-9 and CCR7 expression in predicting the risk of lymph node metastasis in the LSCC patients.

## Material and Methods

**Sample Collection**

A total of 60 tissue samples of LSCC were collected from Department of Anatomical Pathology, Faculty of Medicine, University of Indonesia-Cipto Mangunkusumo Hospital. All of the patients had been diagnosed pathologically as LSCC based on the WHO criteria after being underwent partial/total laryngectomy with neck dissection in the period of December 2017 until December 2019, while none of the patients had neoadjuvant therapy. LSCC patients who were also diagnosed with another type of tumour or had multiple primary cancers (MPCs) and those with incomplete medical records were excluded from this study. The clinicopathological data of the patients were retrospectively collected, including age, gender, size and differentiation of tumour, pathological stage, and lymph node metastasis. The study protocol was approved by the Medical Ethics Committee of Faculty of Medicine, University of Indonesia-Cipto Mangunkusumo Hospital.

**Immunohistochemistry Staining**

Immunohistochemistry staining was performed on 5 μm paraffin-embedded tissue sections. These sections were then deparaffinized using graded xylol and hydrated with different types of alcohol. To expose the studied antigens, the slides were heated using the 0.1 M NaOH citrate buffer (pH 7.0) in an autoclave at 121°C for 20 minutes, then the prepared samples were washed with deionized water for 5 minutes. Endogenous peroxidase was blocked using hydrogen peroxide in 3% methanol for 30 minutes at room temperature, following by washing the slides by Tris Buffer Citrate (TBS) for 5 minutes. Non-specific proteins were blocked with Background Sniper Universal for 15 minutes and then washed again with TBS. Subsequently, the samples were incubated with primary antibody MMP-9 (GeneTex) with a dilution of 1:1000 for one hour and CCR7 (Abcam) with a dilution of 1:500 for overnight.

To evaluate the immunohistochemical expression of MMP-9 and CCR7, two independent pathologists, who were blind to the patients’ clinical data, were involved in this study. They examined the antigens using Leica microscope on well-stained tumors and analyzed the expressions using Image J software. The percentage of MMP-9- and CCR7-positive cells was calculated at 400x magnification in 500 cells in each sample. The expression of MMP-9 and CCR7 was evaluated for each patient using an H-score as a semiquantitative approach by determining the staining intensity as strongly positive (3+), moderate positive (2+), weakly positive (1+), or negative (0). Five hundred cells were examined for each slide to provide a representative score for each sample. The H-score was calculated as (1 × (% cells 1+) + 2 × (% cells 2+) + 3 × (% cells 3+)), resulting in a range of 0–300. 

**Statistical Analysis**

Analysis was performed using IBM SPSS statistics 27.0. The cut-off value and the predictive efficacy of MMP-9 and CCR7 expression in the metastatic status of LSCC was assessed using the receiver operating characteristic (ROC) curves. The association of MMP-9 and CCR7 expression with the other clinicopathological data were evaluated by the Chi-square or Fisher’s exact test. 

## Results

**Fig. 1:**
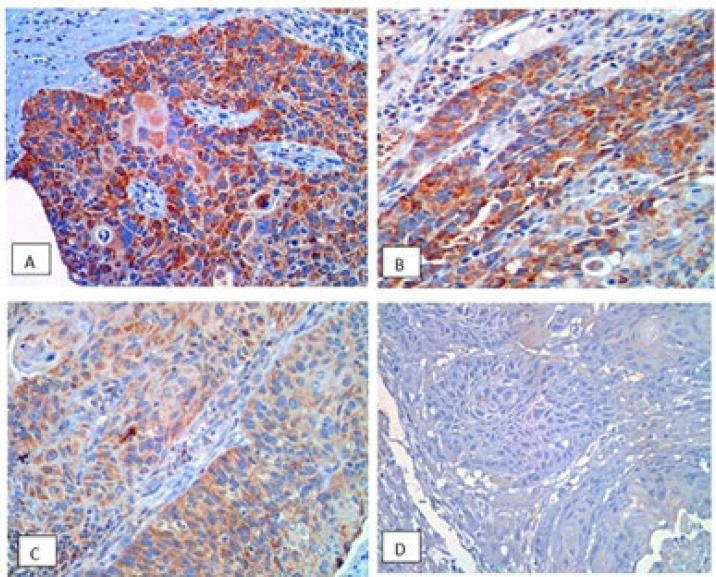
**Matrix metalloproteinase-9 (** **MMP-9) expression in** **laryngeal squamous cell carcinoma** **, 400x magnification**. MMP-9, stained brown in the cytoplasm. A) Expression of MMP-9 with strong intensity; B) Expression of MMP-9 with moderate intensity; C) Expression of MMP-9 with weak intensity; D) Expression of MMP-9 with negative intensity

**Fig. 2:**
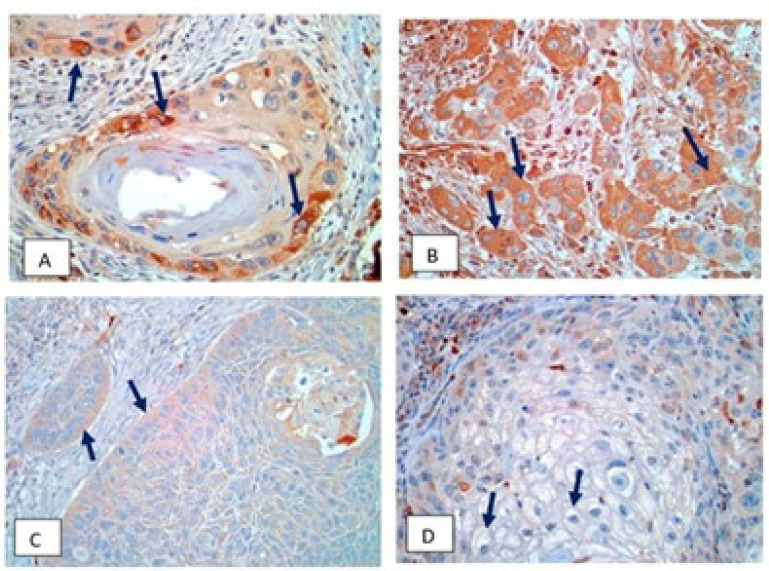
**Chemokine receptor 7 (** **CCR7) expression in** **laryngeal squamous cell carcinoma** **, 400x magnification**. CCR7, stained brown in the cytoplasm; A) Partial expression of CCR7 with strong intensity; B) Expression of CCR7 with moderate intensity; C) Expression of CCR7 with weak intensity; D) Expression of CCR7 with negative intensity

MMP-9 and CCR7 positivity in tumor cells were seen in the cytoplasm and appeared as brown particles (Figures 1 and 2). The expression level of MMP-9 and CCR7 were categorized according to their intensity, comprising strong and moderate, while some cases revealed weak intensity.

**Fig. 3:**
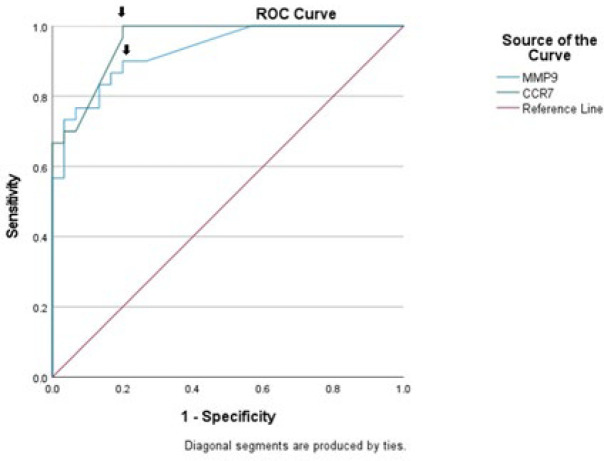
**ROC curve analysis of LSCC metastasis. **Receiver operating characteristic (ROC) curve of MMP-9 and CCR7 expression to predict lymph node metastasis in LSCC. The cut-off between sensitivity and specificity of MMP-9 and CCR7 was best characterized at 100.29 and 98.86, respectively (arrow)

Since this study aimed to underpin the remarkable correlation of MMP-9 and CCR7 expression with the metastatic status of LSCC, we then determined the most valuable MMP-9 and CCR7 expression cut-off points as predictive markers of cervical lymph nodes metastasis in LSCC by applying the ROC curve method for analysis (Figure 3). We then detected that the area under the ROC curve of serum MMP-9 and CCR7 levels predicting cervical lymph nodes metastasis was 0.93 and 0.96, respectively, confirming that both markers had a higher value in predicting the metastasis in LSCC. When the expression of MMP-9 was 100.29, the Youden index was the largest. The sensitivity of MMP-9≥100.29 for predicting the metastasis in the LSCC patients was 90%, and the specificity was 80%. The largest Youden index attributed to the expression of CCR7 was 98.86. Using this value as our cut-off point implies that the sensitivity of CCR7 in predicting the lymph node metastasis was 100%, and the specificity was 80%. The results hinted that the expression of MMP-9 and CCR7 may be a potential marker for predicting the LSCC metastasis.

As Table 1 shows, for metastatic tumor status, the area under curve (AUC) value for ROC curve analysis, based on the expression of MMP-9 and CCR7, was 0.929 (0.869-0.990) and 0.957 (0.913-1.00), respectively. The narrow confidence interval in Table 1 also depicts that MMP-9 and CCR7 had higher accuracy for predicting the LSCC metastasis.

**Table 1:** Area under the curve of ROC logistic regression model

Variable	Area	*P*	Asymptotic 95% Confidence Interval	
			Lower Bound	Upper Bound
MMP-9	0.929	0.000*	0.869	0.990
CCR7	0.957	0.000*	0.913	1.000

* Significant value (*P*<0.05)

According to the classification described in the method, the immunohistochemical analysis clearly showed that high expression of MMP-9 and CCR7 was presented in 78.8% and 83.3% of metastatic LSCC tissues, respectively. These results were significantly different from those of non-metastatic LSCC tissues, which had a high expression of MMP-9 and CCR7 only in 21.2% (7 of 30) and 16.7% (6 of 30), respectively (*P*<0.001) (Table 2).

**Table 2:** Relationship of MMP-9 and CCR7 expression with the metastatic status of LSCC

Variables	N (%)	MMP-9		*P*	CCR7		*P*
		High (n=33)	Low (n=27)		High (n=36)	Low (n=24)	
Metastasis							
Yes	30 (50%)	26 (78.8%)	4 (14.8%)	<0.001*	30 (83.3%)	0 (0%)	<0.001*
No	30 (50%)	7 (21.2%)	23 (85.2%)		6 (16.7%)	24 (100%)	

* Significant value (***P***<0.05)

Dissecting the correlation between clinicopathological features of LSCC patients and the expression of MMP-9 and CCR7 proposed two groups for this study: subjects with high and low expressions of those markers. The results presented in Table 3 reveal that elevated MMP-9 and CCR7 expressions were associated significantly with tumor size (*P*<0.002 and *P*<0.001, respectively). While, other factors, including gender, age, tumor differentiation, lymphovascular invasion, and pathology tumor stage were noticed to have no correlation with the MMP-9 and CCR7 expression. 

**Table 3:** Correlation between MMP-9 and CCR7 expression with clinicopathological features of LSCC

**Variables**	**N (%)**	**MMP-9**		***P***	**CCR7**		***P***
		High (n=33)	Low (n=27)		High (n=36)	Low (n=24)	
**Gender**							
Male	56 (93.3%)	30 (90.9%)	26 (96.3%)	0.405	33 (91.7%)	23 (95.8%)	0.526
Female	4 (6.7%)	3 (9.1%)	1 (3.7%)		3 (8.3%)	1 (4.2%)	
**Age**							
< 50 years	11 (18.3%)	6 (18.2%)	5 (18.5%)	0.360	8 (22.2%)	3 (12.5%)	0.159
50-59 years	17 (28.3%)	7 (21.2%)	10 (37%)		7 (19.4%)	10 (41.7%)	
> 60 Years	32 (53.3%)	20 (60.6%)	12 (44.4%)		21 (58.3%)	11 (45.8%)	
**Tumor Size**							
< 4 cm	36 (60%)	14 (42.4%)	22 (81.5%)	0.002*	15 (41.7%)	21 (87.5%)	<0.001*
> 4 cm	24 (40%)	19 (57.6%)	5 (18.5%)		21 (58.3%)	3 (12.5%)	
**Tumor differentiation**							
Well	13 (21.7%)	4 (12.1%)	9 (33.3%)	0.047	6 (16.7%)	7 (29.2%)	0.250
Moderate-Poor	47 (78.3%)	29 (87.9%)	18 (66.7%)				
**Lymphovascular invasion**							
Yes	6 (10%)	3 (9.1%)	3 (11.1%)	0.795	4 (11.1%)	2 (8.3%)	0.725
No	54 (90%)	30 (90.9%)	24 (88.9%)		32 (88.9%)	22 (91.7%)	
**pT Stage**							
1-2	5 (8.3%)	2 (6.1%)	3 (11.1%)	0.481	2 (5.6%)	3 (12.5%)	0.340
3-4	55 (91.7%)	31 (93.9%)	24 (88.9%)		34 (94.4%)	24 (87.5%)	

* Significant value (*P*< 0.05)

Having interpreted our independent variables as associated with the expression of MMP-9 and CCR7, we then tried to capitalize our study for having detailed information on the statistical correlation of MMP-9 and CCR7 (Table 4). Table below displays the group of LSCC patients with elevated MMP-9 had a high expression of CCR7 (*P*<0.001).

**Table 4:** Association between expression of MMP-9 and CCR7

Variables		CCR7		*P*
		High	Low	
MMP-9	High	28	5	<0.001*
	Low	8	19	

* Significant value (***P***<0.05)

## Discussion

The present study reported that MMP-9 and CCR7 were essential markers for predicting metastasis in LSCC. To corroborate those markers as predicting the LSCC metastasis, we employed a ROC curve analysis to have the cut-off points of MMP-9 and CCR7, then we analysed the area under curve (AUC) to see the significance of both markers in predicting the metastasis. One interesting finding in this study was that the expression of MMP-9 and CCR7 had a significant sensitivity in predicting the metastasis, accounting for 90% and 100%, respectively. The elevated expression of the markers was also attributed significantly to the metastasis event in LSCC (*P*<0.001). Previous studies evaluating the MMP-9 expression associated with the lymph node metastasis of hypopharyngeal carcinoma have revealed that high expression of MMP-9 correlated with the lymph node metastasis and the metastatic lymphatic degree (19). Several studies have also reported that MMP-9 is responsible for a wide range of tumour activities, the majority of which encourage the growth and spread of cancer cells (20, 21). Producing MMP-9 from cancer cells relies on several growth factors and cytokines, which involve the mitogen-activated protein kinases (MAPK) signalling pathway and have been implicated in tumour progression and its spreading (22, 23). Normally, MMP-9 protein is secreted as inactive enzyme (proMMP-9), and this condition is maintained by the bounding of cysteine protein and zinc atom to the active site of proMMP-9. Acidic tumour environment due to the increasing rate of glycolysis, however, might trigger the secretion of Cathepsin K as a member of lysosomal cysteine cathepsin (LCC) subgroup, which competed to disrupt cysteine interaction with zinc in proMMP-9, resulting in active MMP-9 production (24). 

Hypoxia and prostaglandin (PGE2) increase CCR7 expression in cancer cells, leading to the elevation of VEGF-C and VEGF-D expression, which in turn, leads to lymphangiogenesis. Then, the cancer cells enter the lymphatic vessels, though which they migrate to lymph nodes. As lymph nodes have a high expression of CCL21 (CCR7 ligand), the cancer cells with CCR7 expression become trapped and form metastasis there (25). The CCR7-associated lymph node metastasis is also supported by a study of Yu *et al.*, who showed that high expression of CCR7 in lung adenocarcinoma strongly correlates with the incidence of lymph node metastasis through regulating the NFκβ (26, 27). Moreover, evidence suggested that CCR7 might drive immune cell migration into the lymph node, providing a possible role of CCR7 in regulating the cancer cell metastasis (28-30).

Here we also performed statistical analysis to see whether there is a correlation between the expression of MMP-9 and CCR7. Our data suggested that up-regulation of MMP-9 significantly related to the high expression of CCR7. This result confirmed a previous study by Munoz *et al.* in which CCR7 and CCL21 were responsible for upregulating the MMP-9 expression. Additionally, MMP-9 mediated CCL21-driven cell movements (31). Consistent with this result, another study demonstrated that knocking down the CCR7 in human colon cancer cell line vigorously supressed the MMP-9 expression and accordingly the metastasis of colon cancer (32). CCR7 acts as a regulator in the migration of lymphocytes such as dendritic cells, T cells, and B cells to the lymph nodes, while MMP-9 degrades type IV collagen in tumor basement membranes, and extracellular matrix so that tumor cells may invade the surrounding tissues, then metastasis happens. Therefore, CCR7 regulates MMP-9 mediating chemotaxis, and cell migration in LSCC (24). 

Validity from several studies has revealed that MMP-9 and CCR7 could be good predictors and reliable indicators of cancer metastasis. Multiple reports have shown persistent high expression of MMP-9 in various cancer types and its correlation with metastatic processes (33-38). In reviewing the literature on Head and Neck Squamous Cell Carcinoma (HNSCC), the overexpression of MMP-9 upregulated by Galectin-7 interacting with TCF3 might significantly promote lymph node metastasis (39). Furthermore, a previous study evaluated consistent results on the upregulation of CCR7 in predicting locoregional metastasis of human neural stem cells (HNSC). In addition, it has been reported that overexpression of CCR7 in HNSCC tissues facilitated the RhoA/ROCK pathway-mediated migration of tumor cells in HNSCC (40). Data from several resources also reported the consistency of raising CCR7 levels in regulating different types of tumor metastasis (17, 27, 41-46).

The retrospective study design and semi-quantitative immunohistochemistry analysis used in this study to explore the expression of MMP-9 and CCR7 in LSCC was one of the limitations of this study. Moreover, since the samples of this study were collected from only one institute, the problem was consequently with generalizing the results. Furthermore, the absence of a threshold for immunohistochemistry overexpression and the variety of cut-off values between the studies associated with the staining index of MMP-9 and CCR7 in LSCC might lead to the variation of the positive rates and predictive values of MMP-9 and CCR7 expression. This may contribute to the heterogeneity and restrict the clinical implications of MMP-9 and CCR7 expression for LSCC prognosis. 

## Conclusion

The analysis of MMP-9 and CCR7 expression performed in this study has extended our knowledge on the potential of these markers in predicting the lymph node metastasis in LSCC. CCR7 significantly regulated the MMP-9 expression and revealed the highest sensitivity to predict the lymph node metastasis in LSCC compared to the MMP-9 protein. 

## Funding

There was no funding provided to the authors at any stage of the article development or publication.

## Conflict of Interest

There are no conflict of interests declared by the authors.
